# Validation of the 4AT for assessing recovery from delirium in older hospital patients

**DOI:** 10.1093/ageing/afaf166

**Published:** 2025-06-30

**Authors:** Haruno McCartney, Erin Noble, Kali Thompson, Kseniia Fomina, Laura Mesia Guevara, Daniel H J Davis, Jonathan Evans, Susan D Shenkin, Graciela Muniz, Daisy Sandeman, Alasdair M J MacLullich, Zoë Tieges

**Affiliations:** Department of Computer and Information Sciences, University of Strathclyde, Glasgow, UK; Ageing and Health, the Usher Institute, the University of Edinburgh, Edinburgh, UK; Ageing and Health, the Usher Institute, the University of Edinburgh, Edinburgh, UK; Department of Psychological Sciences and Health, University of Strathclyde, Glasgow, UK; Ageing and Health, the Usher Institute, the University of Edinburgh, Edinburgh, UK; Ageing and Health, the Usher Institute, the University of Edinburgh, Edinburgh, UK; Ageing and Health, the Usher Institute, the University of Edinburgh, Edinburgh, UK; MRC Unit for Lifelong Health and Ageing, University College London, UK; School of Health and Wellbeing, University of Glasgow, Glasgow, UK; Ageing and Health, the Usher Institute, the University of Edinburgh, Edinburgh, UK; Advanced Care Research Centre, the Usher Institute, the University of Edinburgh, Edinburgh, UK; Heritage College of Osteopathic Medicine, Ohio University, Athens, USA; Psychiatry, Centre for Dementia Prevention, Royal Edinburgh Hospital, Edinburgh, UK; School of Health in Social Science, the University of Edinburgh, Edinburgh, UK; Ageing and Health, the Usher Institute, the University of Edinburgh, Edinburgh, UK; Ageing and Health, the Usher Institute, the University of Edinburgh, Edinburgh, UK; Department of Computer Science, Glasgow Caledonian University, Glasgow, UK

**Keywords:** 4AT, delirium, recovery, diagnostic accuracy, distress, older people

## Abstract

**Background:**

A crucial part of delirium care is assessing for recovery, yet there are no validated methods for this. The 4AT is a widely used delirium assessment tool, but its performance in assessing recovery remains unstudied. This study evaluated the 4AT’s performance in assessing recovery from delirium.

**Materials and methods:**

In this prospective diagnostic accuracy study, older hospitalised patients (≥70 years) with reference standard delirium on enrolment were assessed 2–4 times over ≤9 days. Paired researchers independently conducted blinded assessments of (i) a reference standard (Diagnostic and Statistical Manual for Mental Disorders, 5th edition), including the Delirium Rating Scale-Revised-98 and neuropsychological tests and (ii) the 4AT (index test, score ≥ 4 positive) plus brief measures of distress and psychotic symptoms.

**Results:**

A total of 120 people with delirium participated [median age 86.3, range 70–99, 67 (55.8%) female and 55 (45.8%) with dementia]. All of them completed the first two assessments, 103 (85.8%) completed three and 69 (57.5%) four. Reference standard delirium was present in 102/120 (85%), 72/103 (69.9%) and 53/69 (76.8%) cases at assessments two to four, respectively. In Receiver Operating Characteristic analyses, the 4AT’s sensitivity for detecting delirium was 0.95 (confidence interval 0.91–0.99), 0.96 (0.91–1) and 0.94 (0.88–1), and specificity was 0.67 (0.13–1), 0.88 (0.71–1) and 1 (1–1) at assessments two to four. In total 18 (15%) participants recovered from delirium. Distress was common in delirium and decreased with recovery.

**Conclusion:**

The 4AT maintains diagnostic accuracy on repeated admissions and may effectively assess delirium recovery in acute hospital settings. Fewer patients than expected recovered within 9 days, suggesting more studies on the natural history of delirium in different settings would be informative.

## Key Points

- Assessing for recovery is an essential part of delirium care.
- There is currently no validated method for assessing recovery from delirium.
- The 4AT maintains good diagnostic accuracy over repeated assessments when compared to a DSM-5 reference standard, making it a suitable tool for assessing delirium recovery.

## Introduction

Delirium is a severe neuropsychiatric syndrome characterised by acute deficits in attention and other domains of cognition, altered level of arousal and psychotic and affective symptoms [1]. Onset is rapid, developing over hours to days, and the presence and severity of symptoms can fluctuate [1]. Delirium is mainly triggered by acute illness, trauma, surgery or drugs and affects up to one in four older patients in acute hospital settings [2, 3]. Natural-history longitudinal studies of delirium are relatively scarce but suggest that delirium typically lasts a few days [4], though up to 20% can persist for weeks or months [5]. Delirium features are heterogeneous and may vary across individuals and over time [6–9].

Accurate and timely identification of delirium is clinically essential due to its association with adverse outcomes, including higher mortality, new institutionalisation, falls, increased risk of dementia and distress for patients and carers [10]. Persistent delirium leads to worse outcomes, such as increased risk of institutionalisation and death [11]. It is therefore crucial to monitor and reassess patients for resolution of delirium, as emphasised in guidelines [12–14], to evaluate treatment effectiveness, manage the risk of complications, inform patients and carers and guide discharge planning.

There is no clear evidence on the best tools for assessing delirium recovery [15, 16] and no methods have been validated for this purpose, as noted in the current guidelines [12, 17]. The existing tools are mostly designed for detecting prevalent delirium at a single assessment, e.g. the 4AT (www.the4at.com) [18], the Single Question in Delirium (SQiD) [19] and the Confusion Assessment Method (CAM) [20], or for identifying incident delirium, e.g. the Recognising Acute Delirium As part of your Routine (RADAR) scale [21].

The 4AT is a brief and practical assessment tool for the detection of delirium. It has been extensively validated [18, 22], detects delirium at expected levels in routine practice [23] and is recommended for use in guidelines [12, 17]. An international survey of clinicians suggested that the 4AT be commonly used to assess recovery from delirium [16], but it has not yet been validated for this purpose.

The main objective of the present study was to determine the diagnostic accuracy of the 4AT in assessing delirium recovery in a representative sample of acute hospitalised patients, against a reference standard based on DSM-5. We also explored additional features of delirium, including distress and psychotic symptoms, with the aim of increasing the understanding of changes in noncognitive symptoms in the context of delirium recovery.

## Method

### Study design

This was a prospective observational diagnostic accuracy study involving patients with delirium recruited between December 2021 and July 2022 from Medicine of the Elderly (MoE), orthopaedic and cardiothoracic wards at the Royal Infirmary of Edinburgh, Scotland, UK. We followed the Standards for Reporting of Diagnostic Accuracy Studies (STARD) guidelines [24]. The Scotland A Research Ethics Committee granted ethical approval (reference 20/SS/0010). The study is registered at ISRCTN48221972.

### Participants

Participants were included if they were aged ≥70 years with current delirium, able to communicate in English and provide either written informed consent directly or proxy consent. Patients were excluded if they were unable to speak English, suffering from an acute life-threatening illness, coma, severe vision/hearing impairment, photosensitive epilepsy or high level of patient or family distress or if no suitable person was available to provide proxy consent if required.

### Measurements and procedures

Recruitment was conducted by trained researchers. Eligible participants were identified and approached through liaison with clinical staff. Informed consent was sought by the researchers. Where potential participants lacked the capacity to consent, an appropriate personal or nominated consultee, guardian, welfare attorney or nearest relative was contacted to provide informed consent.

Paired researchers independently conducted blinded assessments of (i) the reference standard assessment based on DSM-5 criteria for delirium (~20–25 min) and (ii) a 4AT index assessment plus additional brief tests (~5 min). Assessments were conducted on the same day, with the target interval between administrations being 15–60 minutes and a maximum of 3 hours. Reference and index assessments were repeated on up to 4 separate days during the patient’s hospital stay, with no more than 3 days between them.

#### Reference standard assessment

The reference standard assessment measured all the Diagnostic and Statistical Manual for Mental Disorders, 5th edition (DSM-5) criteria for delirium, informed by several information sources, i.e. neuropsychological tests measuring attention, level of arousal, memory, orientation, psychotic features (hallucinations and delusions), informant history and clinical records (Appendix 1).

The neuropsychological assessment tools included the Delirium Rating Scale-Revised-98 (DRS-R98) [25], the Richmond Agitation-Sedation Scale (RASS) [26], three-item recall (immediate and delayed memory), orientation questions (month, time and location) [27], counting backwards from 20 [28], the vigilance ‘A’ task (‘CASABLANCA’) and the DelApp comprising an arousal assessment and attention task [29]. The last two DelApp trials were omitted to shorten test time (maximum score of 8).

#### Index assessment

The index assessment comprised the 4AT [18], which consists of four items: alertness, the Abbreviated Mental Test 4 for cognitive screening, attention testing with months backwards and assessing acute change in mental status obtained from the clinical notes or from an informant. The 4AT is scored from 0 to 12, with a predefined score ≥ 4 indicating delirium.

Additional to the 4AT, several brief assessments were included: days of the week backwards for measuring attention and working memory (score 0–2, higher scores indicate worse cognition); psychotic features via questions on hallucinations and delusions (score 0–2, 1 point per positive answer); assessment of distress using the Quick Distress Assessment Tool (QDAT), newly developed for this study to enhance assessment of delirium-related distress, scoring physical signs (e.g. frowning or looking anxious) and patient responses to ‘How are you feeling?’ and ‘Is anything bothering you?’ (score 0–3, higher scores indicate greater distress); assessment of speech and language via (i) speech production and (ii) speech comprehension (following a command, score 0–2, higher scores indicate greater impairment); and assessment of level of arousal assessed separately from the reference standard via eye opening and eye contact (score 0–4, higher scores indicate greater impairment) (Appendix 2).

The presence of dementia was ascertained through formal diagnosis or clear indicators of diagnosis (including antidementia medications) in the clinical records in discussion with a senior clinician (AMJM) and/or the Informant Questionnaire on Cognitive Decline in the Elderly (IQCODE) using a 3.82 cut-off score [30].

### Grouping of participants

Participants were categorised into four groups, separately for each assessment day—delirium, possible delirium, partial delirium or no delirium—based on the DSM-5 delirium reference standard assessment (Appendix 3). Participants were grouped as having delirium if all DSM-5 criteria were met. Participants were grouped as having *possible* delirium when altered level of arousal, attention deficits or other cognitive impairments were present, but information on their cognitive baseline, fluctuations or acute change in symptoms was missing (e.g. no informant available). Participants with all the information available and some delirium symptoms present but not meeting the full DSM-5 criteria were grouped as having partial delirium [11], which may indicate partial recovery or mild symptoms based on neuropsychological testing and 24-hour information.

### Statistical analyses

The diagnostic accuracy of the 4AT was assessed through cross-sectional comparison of 4AT versus the delirium reference standard at each set of paired assessments using sensitivity, specificity and area under the Receiver Operating Characteristic (ROC) curve (AUC; except for the first assessment, where all the participants had delirium). Changes in 4AT and additional test scores from the first to final assessment (second to fourth, depending on study duration) were analysed using paired samples *t*-tests, both for the entire study sample and for those who recovered by their final assessment.

The internal responsiveness of the 4AT was assessed for the full sample and for those who recovered, using two metrics: (i) effect size and (ii) standardised response mean (SRM) [31]. A logistic regression was performed to predict recovery at the final assessment using 4AT score at baseline, age, dementia diagnosis and the number of assessments as predictors.

A *post hoc* ROC analysis was conducted to assess 4AT performance in those with and those without dementia.

## Results

### Patient characteristics

**Figure 1:**
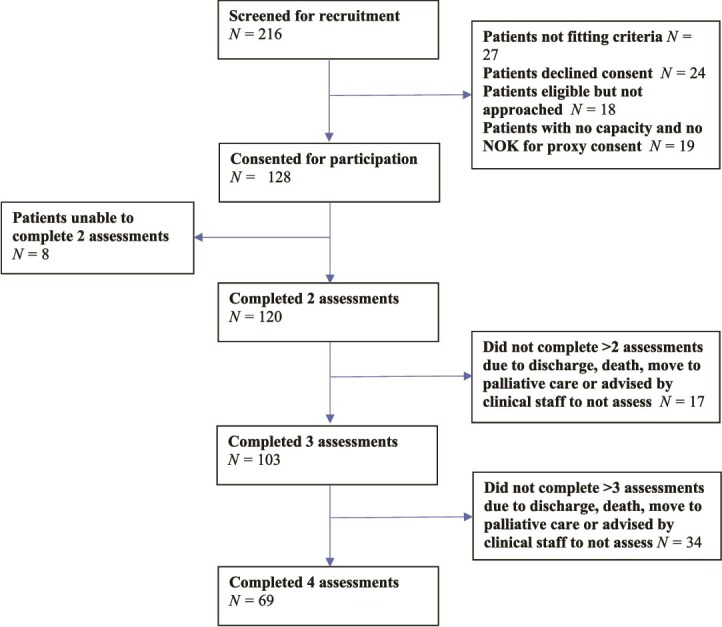
Participant recruitment flow chart.

A total of 189 patients were eligible. Informed consent was obtained from 128 patients or proxies (Figure 1). The participants’ ages ranged from 70 to 99 years (*M* = 86.3, SD = 6.34) and 67 (55.8%) were female. Fifty-five (45.8%) had dementia (Table 1).

**Table 1:** Baseline demographic and clinical patient characteristics.

	Total
**Cases (*N*)**	120
**Sex (female) [number (%)]**	67 (55.8)
**Age (years) (mean ± SD)**	86.3 ± 6.34
**Body mass index (mean ± SD)**	23.33 ± 4.65
**Dementia [number (%)]** Alzheimer’s disease Vascular dementia Mixed Alzheimer’s and vascular Frontotemporal dementia Dementia with Lewy bodies Unspecified	55 (45.8) 17 (14.2) 9 (7.5) 9 (7.5) 1 (0.8) 1 (0.8) 18 (15)
**Psychiatric/Developmental disorder [number (%)]** Clinical depression Anxiety disorder Bipolar with delusions Aphasia	10 (8.3) 5 (4.2)^a^ 3 (2.5) 1 (0.8) 1 (0.8)
**Medications on admission (*n*) (mean ± SD)**	9.02 ± 4.86
**Smoking history [number (%)]** Current smoker Ex-smoker Nonsmoker Not known	11 (9.2) 34 (28.3) 35 (29.2) 40 (33.3)
**IQCODE** ^**b**^ **[number (%)]** Positive (>3.82) Negative (≤3.82) Not completed	76 (63.3) 33 (27.5) 11 (9.2)
**Discharge destination [number (%)]** Home Care home Other hospital Deceased Still in hospital at 12 weeks Not known	46 (38.3) 23 (19.2) 21 (17.5) 20 (16.7) 8 (6.7) 2 (1.7)
**Mortality at 12 weeks [number (%)]**	29 (24.2)
**Length of stay (days) [median (IQR)]**	32 (18.5–61)

^a^One patient with clinical depression also had anxiety disorder. ^b^IQCODE, Informant Questionnaire on Cognitive Decline in the Elderly (score 1–5; score > 3.82 indicates dementia).

Participants were assessed 2–4 times over a period of up to 9 days while in hospital (mean time in study = 5 days, range 2–9). All of them completed two assessments, 103 (85.8%) completed three and 69 (57.5%) completed four. Reasons for attrition included discharge from hospital, transfer to a different ward/hospital, being moved into palliative care, death or being advised by the medical team not to continue (e.g. due to severe patient and/or family distress).

**Figure 2:**
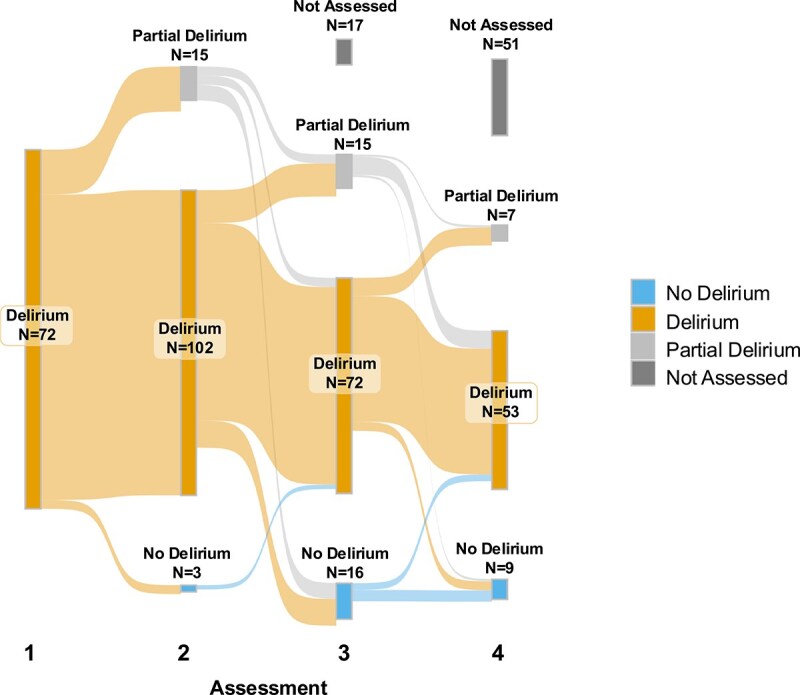
Sankey diagram illustrating the flow of patients through subsequent assessments one to four, with pathways stratified by delirium status (delirium, no delirium or partial delirium). The width of each flow is proportional to the number of patients at each stage, while the diagram also captures changes in grouping across assessments. *Note*. Among participants with partial delirium at the second or third assessment who remained in the study for at least one further assessment, 12 progressed again to full delirium, 8 recovered and 5 continued to exhibit partial delirium.

At the first assessment, all 120 participants had reference standard delirium, with 47% hypoactive according to the RASS, in line with previous studies [32]. At the second assessment, 102/120 (85%) had delirium and 15/120 (12.5%) had partial delirium; at the third, 72/103 (69.9%) and 15/103 (14.5%); and at the fourth, 53/69 (76.8%) and 7/69 (10.1%). No participants were grouped as ‘possible delirium’. Only 18 (15%) patients recovered from delirium (Figure 2).

Based on 4AT scores (≥4), delirium was present in all the participants at the first assessment, 112/120 (93.3%) at the second, 84/102 (82.4%) at the third and 53/69 (76.8%) at the fourth. Across assessments, mean 4AT scores were 8.32 (SD = 2.90, *n* = 347) for participants with delirium, 5.05 (SD = 2.78, *n* = 37) for those with partial delirium and 2.04 (SD = 1.62, *n* = 28) for those without delirium.

### Diagnostic test accuracy of the 4AT on repeat assessments

The AUC of the 4AT for detecting delirium was 0.93 (95% confidence interval: 0.87–0.99) at the second assessment, 0.96 (0.93–0.96) at the third assessment and 0.96 (0.91–0.96) at the fourth assessment (Table 2). Using a cut-off score of ≥4, the 4AT had a sensitivity of 0.95 (0.91–0.99) and specificity of 0.67 (0.13–1) for detecting delirium at the second assessment, a sensitivity of 0.96 (0.91–1) and specificity of 0.88 (0.71–1) at the third assessment and a sensitivity of 0.94 (0.88–1) and specificity of 1 (1–1) at the fourth assessment.

**Table 2:** Diagnostic accuracy of the 4AT for detecting reference standard delirium (cut off ≥ 4) for assessments 2, 3 and 4.

	*n*	Sensitivity	Specificity	Youden’s index
**4AT second assessment (**≥**4) (95% CI)**	Delirium: 102 No delirium: 3	0.95 (0.13–1)	0.67 (0.91–0.99)	0.62
**4AT third assessment (**≥**4) (95% CI)**	Delirium: 72 No delirium: 16	0.96 (0.91–1)	0.88 (0.71–1)	0.84
**4AT fourth assessment (**≥**4) (95% CI)**	Delirium: 53 No delirium: 9	0.94 (0.88–1)	0.100 (1–1)	0.94

*Note.* Numbers are estimates (95% CI). Youden’s index is equal to sensitivity + specificity − 1; a value of 0 indicates that the test has no discriminative value, and a value of 1 indicates a perfect test.

When stratifying by dementia status, the 4AT maintained acceptable diagnostic accuracy for detecting delirium in subgroups with and without dementia (Table 3). For participants with dementia, the AUC was 0.96 (0.93–1), 0.97 (0.93–1) and 0.99 (0.98–1) at assessments two to four, respectively. Sensitivity remained high across assessments at 0.96 (0.91–1), 0.97 (0.92–1) and 0.96 (0.88–1), while specificity was 1 (1–1), 0.75 (0.45–1) and 1 (1–1).

**Table 3:** Diagnostic accuracy of the 4AT for detecting reference standard delirium (cut off ≥ 4) in patients with and without dementia, for assessments 2, 3 and 4.

Group	Assessment	*n*	Sensitivity	Specificity	Youden’s index
**Dementia**	**4AT second assessment (**≥**4) (95% CI)**	Delirium: 51 No delirium: 1	0.96 (0.91–1)	1 (1–1)	0.96
	**4AT third assessment (**≥**4) (95% CI)**	Delirium: 36 No delirium: 8	0.97 (0.92–1)	0.75 (0.45–1)	0.72
	**4AT fourth assessment (**≥**4) (95% CI)**	Delirium: 24 No delirium: 4	0.96 (0.88–1)	1 (1–1)	0.96
**No dementia**	**4AT second assessment (**≥**4) (95% CI)**	Delirium: 51 No delirium: 2	0.94 (0.88–1)	0.5 (0–1)	0.83
	**4AT third assessment (**≥**4) (95% CI)**	Delirium: 36 No delirium: 8	0.94 (0.87–1)	1 (1–1)	0.92
	**4AT fourth assessment (**≥ **4) (95% CI)**	Delirium: 29 No delirium: 5	0.93 (0.84–1)	1 (1–1)	0.83

*Note*. Numbers are estimates (95% CI). Youden’s index is equal to sensitivity + specificity − 1; a value of 0 indicates that the test has no discriminative value, and a value of 1 indicates a perfect test.

For participants without dementia, the AUC was 0.87 (0.73–1), 0.99 (0.97–1) and 0.94 (0.85–1), respectively. Sensitivity remained high at 0.94 (0.88–1), 0.94 (0.87–1) and 0.93 (0.84–1), whereas specificity varied from 0.5 (0–1) at the second assessment to 1 (1–1) at both the third and fourth assessments.

### Responsiveness to change

Eighteen (15%) participants fully recovered from delirium according to the reference standard and 26 (22%) according to the 4AT (score <4). The 4AT scores improved from the first to the last assessment in the entire study sample [*t* = 6.00, df = 119, *P* < .001; mean 4AT scores at the first and last assessments were 8.6 (SD = 2.60) and 6.6 (SD = 3.76), respectively]. Among the 18 who recovered, the mean 4AT scores improved from 7.1 (SD = 2.07) to 1.7 (SD = 1.50) (*t* = 9.06, df = 17, *P* < .001) (Appendix 4).

The 4AT showed moderate internal responsiveness in the overall sample (effect size = −0.69, SRM = −0.55). Among those who recovered, responsiveness was strong (effect size = −2.60, SRM = −2.14), indicating marked improvement in 4AT scores. Lower baseline 4AT scores predicted recovery (*β* = −0.27, SE = 0.12, *z* = −2.19, *P* = .03), with each 1-point decrease score increasing recovery odds by ~32% (variance inflation factor values < 1.1).

### Additional features of delirium

At the first assessment (all delirium), 64% (76/118) of the patients experienced distress (QDAT > 0), with 25% (29/118) having moderate-to-severe distress (QDAT = 2–3). By the final assessment, 53% (62/118) were still distressed, with 20% (24/118) experiencing moderate-to-severe distress. Across all the assessments, distress was present in 63% (217/343) of delirium cases compared to 25% (7/28) in those who had recovered. Moderate-to-severe distress occurred only in delirium (26%, 89/343). Patients who fully recovered from delirium during the study showed a reduction in distress over time (*t* = 2.70, df = 17, *P* < .05) (Appendix 5).

Psychotic symptoms were reported by 40% (44/110) of the participants at the first assessment (all delirium) and 24% (28/116) at their last assessment. Across all the assessments, psychotic symptoms were more common in patients with delirium (37%, 117/319) than in those without delirium (21%, 6/28). Patients who fully recovered showed a reduction in psychotic symptoms between the first and last assessments (*t* = 2.36, df = 17, *P* < .05).

At the first assessment, 75% (90/120) of the patients (all delirium) could not complete the days of the week backwards without errors. By their final assessment, this proportion had decreased to 63% (76/120). Among those who did not recover from delirium, 72% (65/90) still failed the task by their final assessment. In contrast, patients who fully recovered showed a significant improvement between the first and last assessments (*t* = 3.29, df = 17, *P* < .01).

Arousal impairment was seen in 61% (73/120) of the patients at the first assessment (all delirium), and decreased to 49% (59/120) at their last assessment, with no significant improvement observed in those who fully recovered (*t* = 1.84, df = 17, *P* = .08). Speech and language ability was impaired in 34% (41/120) of the participants at the first assessment and remained stable throughout the assessments, with no improvement seen in recovered patients.

## Discussion

The 4AT retained good diagnostic accuracy for delirium across repeated assessments, with sensitivities between 0.94 and 0.96 and specificities between 0.67 and 1 compared to the reference standard. The relatively low specificity of 0.67 at the second assessment was likely due to the small number of delirium-negative participants [*n* = 3 (2.5%)]. The 4AT had acceptable performance in participants with and without dementia across assessments, which suggests it can measure delirium recovery even in patients with dementia. The 4AT showed moderate responsiveness overall and high responsiveness in recovered patients, indicating sensitivity to changes in delirium status.

This study adds to the limited literature on delirium features over time in general hospital settings. A key finding was that fewer patients recovered than expected [*n* = 18 (15%)], despite a mean study duration of 5 days (range 2–9) suggesting that delirium may last longer in this population than the 3–4 days reported in some studies [4, 11, 33]. This may, in part, reflect the comprehensive nature of the DSM-based assessment used, which considers a wide range of symptoms (cognitive, psychiatric and affective) informed by multiple sources of information. In contrast, studies relying on brief delirium tools or clinical judgement alone may have underestimated delirium duration, especially in milder presentations.

Distress (measured with the QDAT) was more common in patients whose delirium persisted than in those whose delirium resolved, occurring in over half of the delirium cases, with 26% having moderate-to-severe distress. Patients who recovered showed a decrease in distress and psychotic symptoms, alongside cognitive improvement. There was no change in speech and language. These findings suggest that repeated assessment of distress, psychotic features and cognitive function may aid in monitoring delirium recovery.

### Clinical implications of assessing for delirium recovery

Delirium significantly impacts patients, carers and various aspects of care. It is both a marker and potential accelerator of existing dementia and a risk factor for new dementia [34–36]. Delirium also has a financial impact on healthcare systems, with a recent study linking delirium to increased costs in hospital, post discharge and in long-term care facilities [37]. Discharging patients with unresolved delirium is sometimes feasible but may pose significant risks, and determining whether a patient has delirium is essential in managing these risks; in addition, continuing management at home should include ongoing delirium assessments to ensure that the condition is resolving [38].

The 4AT is used routinely in clinical practice to detect delirium [17] and to assess recovery [16]. Its good diagnostic accuracy when repeatedly administered makes it a strong candidate as a clinical tool for assessing recovery from delirium. Scores of the 4AT indicating delirium may help predict recovery, consistent with studies showing that early changes in frailty or dementia severity are linked to better prognosis, emphasising the value of early recovery assessment in outcome prediction [39, 40].

### Strengths and limitations

The present study has several strengths. The sample was representative of older patients with delirium in acute settings, with around half having dementia [37] and half being hypoactive [31]. The participants were assessed at least twice; 86% were assessed three times and 58% four times. This study adds to the relatively small literature of longitudinal studies of delirium, reporting both delirium presence and features over time. A detailed reference standard was used, including neuropsychological testing to capture delirium symptom domains and inform DSM-5 criteria. Researchers conducting the reference standard and index assessments were blinded to each other’s scores to ensure objectivity. Some tools (e.g. DRS-R98) may return a delirium-negative result score in patients unable to engage with cognitive testing (e.g. hypoactive patients), or a score indicating no impairment; therefore additional tools (e.g. vigilance and DelApp) were used.

The study included participants with dementia and pre-existing cognitive impairment, with 46% having dementia. ROC analyses showed that the 4AT has good diagnostic accuracy across assessments in those with and those without dementia, indicating its sensitivity to delirium fluctuations, even in those with dementia. While differentiating delirium from dementia can be challenging, the comprehensive reference standard likely minimised misdiagnoses.

Some limitations should be acknowledged. The diagnostic accuracy of the 4AT may have been impacted by the relatively low number of people whose delirium had resolved at repeat assessments. The finding that fewer participants recovered than expected aligns with literature showing delirium can persist beyond a few days [5]. Dementia is underdiagnosed in the UK population [41], so some cases may have been missed despite using IQCODE and clinical indicators of diagnosis (including antidementia medications). The QDAT, developed for this study in the absence of suitable tools, needs further validation, though the high levels of distress detected suggest this is an important aspect of delirium assessment and care. Discrepancies between the reference standard and 4AT may reflect differences in time frames: the DRS-R98 scores symptoms over 24 hours, while the 4AT assesses alertness and cognition at the time of evaluation. Symptom fluctuation may not have been fully captured by the DSM reference if clinical notes lacked sufficient 24-hour details, potentially leading to misclassification of recovery and perceived recrudescence. Patients were not followed beyond 9 days to balance data capture with minimising patient loss due to discharge, relocation or death.

### Future research

This study shows that the 4AT can be used for repeat delirium assessment over short timescales. Future research should investigate its diagnostic accuracy over longer periods, such as in persistent delirium, and assess its utility in other populations, e.g. younger age groups and critical care patients. The predictive validity of recovery trajectories assessed with the 4AT and long-term outcomes, including dementia, requires further exploration to identify individuals at higher risk of cognitive decline following delirium. Frailty is a key factor for future research on delirium recovery, as it is a well-established delirium risk factor, and its combination with delirium may indicate a particularly poor prognosis [42, 43].

Repeated use of the same cognitive assessments raises concerns about acceptability to patients and practice effects, which require further study. Alternative assessment versions or subdomains of the 4AT may address these issues, but this requires further validation. The value of assessing features not captured by tools like the 4AT, such as distress and psychotic symptoms, also warrants further investigation.

## Conclusion

Due to its good diagnostic accuracy, its internal responsiveness and its clinical applicability, the 4AT may be an effective tool for measuring delirium recovery in acute older inpatients. Standardisation of the assessment of delirium recovery may result in reduction in discharge with unresolved delirium and improve delirium care overall.

## Supplementary Material

Supplementary_materials_afaf166

## Data Availability

The data that support the findings of this study are available from the corresponding author upon reasonable request.
