# Correction to: Validation of the 4AT for assessing recovery from delirium in older hospital patients

**DOI:** 10.1093/ageing/afag017

**Published:** 2026-01-28

This is a correction to: Haruno McCartney, Erin Noble, Kali Thompson, Kseniia Fomina, Laura Mesia Guevara, Daniel H J Davis, Jonathan Evans, Susan D Shenkin, Graciela Muniz, Daisy Sandeman, Alasdair M J MacLullich, Zoë Tieges, Validation of the 4AT for assessing recovery from delirium in older hospital patients, *Age and Ageing*, Volume 54, Issue 6, June 2025, https://doi.org/10.1093/ageing/afaf166

An **Acknowledgement** section was added to the original publication of the article, with the statement: “The authors acknowledge that Erin Noble and Haruno McCartney contributed equally as joint first authors of this work”.

