# Supplementary material for: Validation of the 4AT for assessing recovery from delirium in older hospital patients
Source: Age Ageing. 2025 Jun 30;54(6):afaf166. doi: 10.1093/ageing/afaf166 (PMC12207212; doi:10.1093/ageing/afaf166)
Supplement: Supplementary_materials_afaf166 [file supplementary_materials_afaf166.docx]

**Validation of the 4AT for assessing recovery from delirium in older hospital patients.**

**List of contents**

**Appendix 1.** Case Report Form for the DSM-5 delirium reference standard assessment (Assessment 1).

**Appendix 2**. Case Report Form for index assessment including the 4AT (Assessment 1).

**Appendix 3**. DSM-5 structured reference standard flowchart for delirium.

**Appendix 4**. Supplementary Figure S1. 4AT scores across assessments for 18 participants who recovered from delirium (ie. grouped as 'no delirium') by their last assessment.

**Appendix 5**. Supplementary Table S1. Reference and index assessment scores for assessments 1, 2, 3 and 4.

**Appendix 1**. Case Report Form for the DSM-5 delirium reference standard assessment (Assessment 1).

**Case Report Form – Reference Standard Assessment**

| **Participant ID** | **Site** | **Visit No** | **Date of visit** |
| --- | --- | --- | --- |
| ______________ | Edinburgh, RIE | Baseline ☐  2☐ 3☐ 4☐ | ___/___/____ |

***To be completed by Researcher 1***

**Assessment Details**

**Eligibility screening checklist completed: Yes ☐ No ☐**

**Patient or legal proxy has consented: Yes ☐ No ☐**

**Start time: ________**

**Finish time: ________**

**Inter-rater assessment: Yes □ No □ Initials of Researcher: _______**

*Note: Use the specified wording of the questions when carrying out assessments*

**INTRODUCTION**

Introduce yourself and ask the patient what they would like to be called.

1. **“Would you be able to tell me your full name?”**
2. **“What is your date of birth?”**

(No scoring)

**SHORT-TERM MEMORY: Three-Item Recall**

Assessment Version: 1 □ 2 □ 3 □ 4 □

Show the participant the three images and say **“Please look at these pictures. This is a *lemon, a key and a ball.*”** (NB: different items for each assessment version)

Then remove the images and ask the participant **“Could you now tell me again, what pictures you have just seen?”** (Score as short-term memory)

After participant repeats the items, say **“Try to remember them because I am going to ask you again later”.**

*Score only the first trial (but repeat 3 times if necessary to help encoding).*

**Three-Item Recall (0 to 3)**

Recalls 3/3 items; short-term memory is intact (0 points) □

Recalls 2/3 items; may be able to recall third item after category cueing (1 point) □

Recalls 1/3 items; may be able to recall other items after category cueing (2 points) □

Recalls 0/3 items (3 points) □

**Three-Item Recall (STM) Total Score: ____ /3**

**Three-Item Recall (STM) Assessment Complete:** **Yes** □ **No** □ **UTA** □

**ORIENTATION Assessment**

**1. What month is it now?**

No answer given or incorrect month (1 point) □

Correct month stated (0 points) □

**2. Without looking at the clock, could you tell me what time it is to the nearest hour?**

No answer given or incorrect time (1 point) □

Correct time given to the nearest hour (0 points) □

**3. Do you know where we are right now?**

No answer given or incorrect location (1 point) □

Correct location given (0 points) □

**Orientation Assessment Total Score: ____ /3**

**Orientation Assessment Complete:** **Yes** □ **No** □ **UTA** □

**ATTENTION: Counting Backwards Test (20 to 1)**

**“Could you please count backwards from 20 down to 1?”**

No mistakes (0 points) □

1 mistake (2 points) □

2+ mistakes, no response or UTA (4 points) □

**Counting Backwards Total Score: ____ /4**

**Counting Backwards Assessment Complete:** **Yes** □ **No** □

**ATTENTION: CASABLANCA**

**“I am going to read you a series of 10 letters. Whenever you hear the letter ‘A’, indicate by squeezing my hand.”** Read letters in a normal tone, 3 seconds apart.

*Tick when patient squeezes hand.*

**C** □ **A** □ **S** □ **A** □ **B** □ **L** □ **A** □ **N** □ **C** □ A □

Errors are counted (+1 point) when patient fails to squeeze on the letter “A” and when the patient squeezes on any letter other than “A”.

**CASABLANCA Total Score: ____ /10**

**CASABLANCA Assessment Complete:** **Yes** □ **No** □ **UTA** □

**ATTENTION: DelApp Assessment**

Assessment Version: 1 □ 2 □ 3 □ 4 □ 5 □

***Part 1: Assessment of level of arousal***

**1a)** Is the patient responsive or arousable, or does the patient open eyes for more than 10 seconds to speech (say their name) or touch on shoulder? Yes (1 point) □ No (0 points) □

**1b)** Does the patient open eyes briefly (less than 10 seconds) to speech or touch on the shoulder? (1 point) Yes (1 point) □ No (0 points) □

**2)** Can the patient say their name, or (if not) obey a one stage command, for example lifting one arm? (1 point) Yes (1 point) □ No (0 points) □

**3)** Can the patient follow an object with their eyes for 5 seconds? (1 point)

Yes (1 point) □ No (0 points) □

Total Arousal Score: ____ /4 (If score is 3 or above proceed to Part 2)

***Part 2: Assessment of visual ability (pre-test)***

**“In a moment you will see a white star on the screen of the phone. Can you let me know when you see this star? Is that clear?”**

Accurately identified shape? Yes □ No □ N/A □ **(If no then end the assessment now)**

***Part 3: Sustained Visual Attention Task***

Explain to the participant [while pointing at the smartphone screen]:

**“Every now and then a white star will appear on the screen. I want you to keep looking at the screen and count how many stars you see. You can count in your head [silently]. You should keep counting until I ask you how many stars you saw. Is that clear? Are you ready? Here we go.”**

| **Practice (trial 1)** | **Trial 2** | **Trial 3** |
| --- | --- | --- |
| Correct □ | Correct (1 point) □ | Correct (1 point) □ |
| Incorrect □ | Incorrect (0 points) □ | Incorrect (0 points) □ |
| No answer □ | No answer (0 points) □ | No answer (0 points) □ |
| Not assessed □ | Not assessed (0 points) □ | Not assessed (0 points) □ |
| Participant response | Participant response | Participant response |

**“As before, every now and then a white star will appear on the screen. I want you to keep looking at the screen and count how many stars you see. You will also see some small triangles appearing on the screen. Please try ignoring these triangles and just keep counting the stars as before. You should keep counting until I ask you how many stars you saw. Is that clear? Are you ready? Here we go.”**

| **Trial 4** | **Trial 5** |
| --- | --- |
| Correct (1 point) □ | Correct (1 point) □ |
| Incorrect (0 points) □ | Incorrect (0 points) □ |
| No answer (0 points) □ | No answer (0 points) □ |
| Not assessed (0 points) □ | Not assessed (0 points) □ |
| Participant response | Participant response |

Total Attention Score: ____ /4

**DelAppTotal Score: ____ /8**

**DelApp Assessment Complete:** **Yes** □ **No** □ **UTA** □

**DRS-R98 (Patient interaction to inform sections 1-3 & 13)**

Score in DRS-R98 below.

**Section 1: Sleep-wake cycle disturbance**

Ask about sleeping patterns: **“Have you been sleeping well?”; “How have the nights been here?”**

*Notes:* ___________________________________________________________________

_________________________________________________________________________

**Section 2: Perceptual disturbances and hallucinations**

Elicit any hallucinations by asking: **“Sometimes when people are in hospital, they see or hear things that are not there, due to medication or tiredness – has this happened to you?”**

*Notes:* ___________________________________________________________________

_________________________________________________________________________

**Section 3: Delusions**

Elicit any delusions by asking: **“Is there anything that has been worrying you since coming to the hospital?”; “Do you feel frightened by anything or anyone?”**

*Notes:* ___________________________________________________________________

_________________________________________________________________________

**Section 13: Visuospatial Ability**

To get an account of the patient’s visuospatial ability, ask: **“Does the room look normal to you?”; “Is anything moving that shouldn’t be?”; “Are the windows and doors in the right place?”**

*Notes:* ___________________________________________________________________

_________________________________________________________________________

**LONG-TERM MEMORY: Three-Item Recall**

Assessment Version: 1 □ 2 □ 3 □ 4 □

**“Remember the 3 pictures I showed you earlier? Would you be able to tell what they were?”**

**Three-Item Recall (0 to 3)**

Recalls 3/3 items (0 points) □

Recalls 2/3 items (1 point) □

Recalls 1/3 items (2 points) □

Recalls 0/3 items (3 points) □

**Three-Item Recall (LTM) Total Score: ____ /3**

**Three-Item Recall (LTM) Assessment Complete:** **Yes** □ **No** □

**END OF PATIENT INTERACTION**

**SEDATION: Richmond Agitation Sedation Scale (RASS)**

Score level of sedation directly after bedside visit (-5 to 4).

**Combative:** Overtly combative or violent; immediate danger to staff (4 points) □

**Very agitated:** Pulls on or removes tube(s) or catheter(s) or has aggressive behavior toward staff (3 points) □

**Agitated:** Frequent non-purposeful movement or patient–ventilator dyssynchrony (2 points) □

**Restless:** Anxious or apprehensive but movements not aggressive or vigorous (1 point) □

**Alert and calm:** (0 points) □

**Drowsy:** Not fully alert, but has sustained (more than 10 seconds) awakening, with eye contact, to voice (-1 point) □

**Light sedation:** Briefly (less than 10 seconds) awakens with eye contact to voice

(-2 points) □

**Moderate sedation:** Any movement (but no eye contact) to voice (-3 points) □

**Deep sedation:** No response to voice, but any movement to physical stimulation

(-4 points) □

**Unrousable:** No response to voice or physical stimulation (-5 points) □

**RASS Total Score: ____ /4**

**RASS Assessment Complete:** **Yes** □ **No** □

**AROUSAL: Observational Scale of Level of Arousal (OSLA)**

**Part 1: Eye opening**

Open on arrival and remain so, under patient’s control, outlasts stimulus (0 points) □

Open on arrival but close if stimulus removed (1 point) □

Open to voice but then outlast stimulus (1 point) □

Open to voice but close if stimulus removed (2 points) □

Open to gentle physical stimulation (squeezing hand, shaking shoulder) (3 points) □

Open to pain only (4 points) □

No eye opening (5 points) □

*Total Eye Opening Score: ____ /5*

**Part 2: Eye contact**

Spontaneously makes and holds eye contact appropriately (0 points) □

Drowsy and makes eye contact to command but can’t hold it for very long (1 point) □

Alert but eyes wandering, some appropriate eye contact (1 point) □

Alert but eyes wandering, little or no appropriate eye contact (1 point) □

Drowsy but makes brief eye contact (2 points) □

Eyes will / are open but no eye contact (3 points) □

*Total Eye Contact Score: ____ /3*

**Part 3: Posture (take into account weakness due to stroke or neurological disease etc.)**

Sitting in chair or up in bed, holding appropriate posture (0 points) □

Slumped in chair or bed but attempts to sit upright and sustain posture on request (1 point) □

Slumped in chair or bed and unable to sustain posture (2 points) □

Lying in bed and unable or no response to request to sustain posture (3 points) □

*Total Posture Score: ____ /3*

**Part 4: Movement**

Moves purposefully with no restless or agitated movements (0 points) □

Occasional or mild restless or fidgety movements; no aggressive or vigorous movements (1 point) □

Reduced frequency of movement, mildly slowed up (1 point) □

Frequently restless/fidgety but no aggressive/vigorous movement (2 points) □

Moderately reduced frequency and speed of movement, interfering with assessment or self-care (2 points) □

Aggressive or vigorous, recent pulling out of lines (3 points) □

Overtly combative, violent (4 points) □

Severely reduced frequency and speed of movement, few spontaneous movements (4 points) □

*Total Movement Score: ____ /4*

**OSLA Total Score: ___ /15**

**OSLA Assessment Complete:** **Yes** □ **No** □

**SEVERITY: Delirium Rating Scale (DRS-R98)**

**Part 1: Sleep-Wake Cycle Disturbance**

Not present (0 points) □

Mild sleep disturbance at night or occasional drowsiness during the day (1 point) □

Moderate disorganisation of sleep-wake cycle (e.g., falling asleep during

conversations or very little night-time sleep) (2 points) □

Severe disruption of sleep-wake cycle (e.g., day-night reversal of sleep-wake cycle or severe circadian fragmentation etc.) (3 points) □

*Total Sleep-Wake Cycle Disturbance Score: ____ /3*

**Part 2: Perceptual Disturbances and Hallucinations**

Not present (0 points) □

Mild perceptual disturbances (e.g., patient not able to discriminate dreams from reality) (1 point) □

Illusions present (2 points) □

Hallucinations present (3 points) □

*Total Perceptual Disturbances and Hallucinations Score: ____ /3*

**Part 3: Delusions**

Not present (0 points) □

Mildly suspicious, hyper-vigilant, or preoccupied (1 point) □

Unusual or overvalued ideation that does not reach delusional proportions or could be plausible (2 points) □

Delusional (3 points) □

*Total Delusions Score: ____ /3*

**Part 4: Lability of Affect**

NB: Rate outward presentation of emotions, not any description of what the patient feels.

Not present; emotions are controlled (0 points) □

Affect somewhat altered or incongruent to situation; changes over the course of hours; emotions are mostly under self-control (1 point) □

Affect is often inappropriate to the situation and intermittently changes over the course of minutes; emotions are not consistently under self-control, though they respond to redirection by others (2 points) □

Severe and consistent disinhibition of emotions; affect changes rapidly, is inappropriate to context, and does not respond to redirection by others (3 points) □

*Total Lability of Affect Score: ____ /3*

**Part 5: Language**

Normal language (0 points) □

Mild impairment inc. word-finding difficulty or naming/fluency problems (1 point) □

Moderate impairment inc. comprehension difficulties or deficits in meaningful communication (semantic content) (2 points) □

Severe impairment inc. nonsensical semantic content, word salad, muteness, or severely comprehension (3 points) □

*Total Language Score: ____ /3*

**Part 6: Thought Process Abnormalities**

Normal thought processes (0 points) □

Tangential or circumstantial (1 point) □

Associations may be loosely connected, but largely comprehensible (2 points) □

Associations loosely connected most of the time (3 points) □

*Total Thought Process Abnormalities Score: ____ /3*

**Part 7: Motor Agitation**

No restlessness or agitation (0 points) □

Mild restlessness of gross motor movements or mild fidgetiness (1 point) □

Moderate motor agitation including dramatic movements of the extremities, pacing, fidgeting, removing intravenous lines, etc. (2 points) □

Severe motor agitation, e.g., combativeness or need for restraints/seclusion (3 points) □

*Total Motor Agitation Score: ___ /3*

**Part 8: Motor Retardation**

NB: Do not rate components of retardation that are caused by parkinsonian symptoms. Do not rate drowsiness or sleep.

No slowness of voluntary movements (0 points) □

Mildly reduced frequency, spontaneity or speed of motor movements, to the degree that may interfere somewhat with the assessment (1 point) □

Moderately reduced frequency, spontaneity or speed of motor movements to the degree that it interferes with participation in activities or self-care (2 points) □

Severe motor retardation with few spontaneous movements (3 points) □

*Total Motor Retardation Score: ____ /3*

**Part 9: Orientation**

*See Orientation Assessment and Introduction Qs to score item 9*

Oriented to person, place and time (0 points) □

Disoriented to time (e.g., by more than 2 days or wrong month or wrong year) or to place (e.g., name of building, city, state), but not both (1 point) □

Disoriented to time and place (2 points) □

Disoriented to person (3 points) □

*Total Orientation Score: ____ /3*

**Part 10: Attention**

*Use attention tests (Counting backwards from 20 and SAVEAHAART) to score item 10 and also consider overall level of attention during interaction.*

Alert and attentive (0 points) □

Mildly distractible or mild difficulty sustaining attention, but refocuses with cueing; on formal testing makes only minor errors and is not significantly slow (1 point) □

Moderate inattention with difficulty focusing and sustaining attention; on formal testing makes numerous errors and requires prodding to focus/finish task (2 points) □

Severe difficulty focusing and/or sustaining attention, with many incorrect or incomplete responses or inability to follow instructions; distractible by other noises or events in the environment (3 points) □

*Total Attention Score: ____ /3*

**Part 11: Short-Term Memory**

*See 3-item recall (first completed assessment) to score item 11*

Recalls 3/3 items; short-term memory is intact (0 points) □

Recalls 2/3 items (1 point) □

Recalls 1/3 items (2 points) □

Recalls 0/3 items (3 points) □

*Total Short-Term Memory Score: ____ /3*

**Part 12: Long-Term Memory**

*See 3-item recall (last completed assessment) to score item 12*

NB: Test item recall after at least 5 minutes, anywhere up to 15-20 minutes.

No significant long-term memory deficits (0 points) □

Recalls 2/3 items and/or has minor difficulty recalling details of other long-term information (1 point) □

Recalls 1/3 items and/or has moderate difficulty recalling other long-term information (2 points) □

Recalls 0/3 items and/or has severe difficulty recalling other long-term information (3 points) □

*Total Long-Term Memory Score: ____ /3*

**Part 13: Visuospatial Ability**

*See visuospatial Qs to score item 13*

NB: Take into account any visual impairments that may affect performance and/or patient’s navigation of environment.

No impairment (0 points) □

Mild impairment such that overall design and most details or pieces are correct; and/or little difficulty navigating in his/her surroundings (1 point) □

Moderate impairment with distorted appreciation of overall design and/or several errors of details or pieces; and/or needing repeated redirection to keep from getting lost in a newer environment despite, trouble locating familiar objects in immediate environment (2 points) □

Severe impairment on formal testing; and/or repeated wandering or getting lost in environment (3 points) □

*Total Visuospatial Ability Score: ____ /3*

**Part 14: Temporal Onset of Symptoms**

NB: Rate the acuteness of onset of the initial symptoms of the delirium episode being currently assessed, not their total duration.

No significant change from usual or longstanding baseline behavior (0 points) □

Gradual onset of symptoms, occurring over a period of weeks to a month (1 point) □

Acute change in behaviour or personality occurring over days to a week (2 points) □

Abrupt change in behaviour occurring over a period of hours to a day (3 points) □

*Total Temporal Onset Score: ____ /3*

**Part 15: Fluctuation of Symptom Severity**

No symptom fluctuation (0 point) □

Symptom intensity fluctuates in severity over hours (1 point) □

Symptom intensity fluctuates in severity over minutes (2 points) □

*Total Fluctuation of Symptom Severity Score: ____ /2*

**Part 16: Physical Disorder**

NB: Many patients have physiological, medical or pharmacological problems but they may or may not have causal relationship to the symptoms being rated; rate only those which can be specifically attributed to have caused the symptoms being assessed.

None present or active (0 points) □

Presence of any physical disorder that might affect mental state (1 point) □

Drug, infection, metabolic disorder, CNS lesion or other medical problem that can be implicated in causing the altered behaviour or mental state (2 points) □

*Total Physical Disorder Score: ____ /2*

**DRS-R98 Total Score: ___ /46**

**DRS-R98 Assessment Complete:** **Yes** □ **No** □

**Total Score**

**Reference Standard Assessments Total Score: ___ /96**

**Assessment Complete:** **Yes** □ **No** □

| **Researcher name** | **Researcher signature** |
| --- | --- |
| **Date** ____/____/____ | |

**Additional Comments**

________________________________________________________________________________________________________________________________________________________________________________________________________________________________________________________________________________________

**Consideration of Adverse Events**

Adverse events were considered by the researcher.

**Signature:** ______________________

Tick to indicate if any events occurred during the assessment.

| **AE** | **Yes** □ | **No** □ |
| --- | --- | --- |
| **SAE** | **Yes** □ | **No** □ |
| **ADE** | **Yes** □ | **No** □ |
| **SADE** | **Yes** □ | **No** □ |

**If applicable, please refer to the Adverse Event Log, located in the Investigator Site File Volume II Section 5.2, for additional information regarding this event.**

**Appendix 2**. Case Report Form for index assessment including the 4AT (Assessment 1).

**Case Report Form – Index Assessment**

| **Participant ID** | **Site** | **Visit No** | **Date of visit** |
| --- | --- | --- | --- |
| ______________ | Edinburgh, RIE | Baseline ☐  2☐ 3☐ 4☐ | ___/___/____ |

***To be completed by Researcher 2***

**Assessment Details**

**Eligibility screening checklist completed: Yes ☐ No ☐**

**Patient or legal proxy has consented: Yes ☐ No ☐**

**Start time: ________**

**Finish time: ________**

**Inter-rater assessment: Yes □ No □ Initials of Researcher: _______**

*Note: Use the specified wording of the questions when carrying out assessments*

**4AT**

**Part 1: Assessment of Alertness**

**1a)** Is the patient responsive or arousable, or if the patient is asleep, do they wake with speech or a gentle touch on their shoulder? Ask them to state their name and their address to assist with rating.

Normal (fully alert, but not agitated, through assessment) (0 points) □

Mild sleepiness for <10 seconds after waking, then normal (0 points) □

Clearly abnormal (4 points) □

Total Arousal Score: ____ /4

**Part 2: Abbreviated Mental Test-4 (AMT-4)**

**2a)** Ask the patient **“What is your date of birth?”; “How old are you?”; “Would you be able to tell me the name of this building?”; “What year is it?”** (To assess for orientation to age, date of birth, place (name of the building specifically) and current year).

No mistakes (0 points) □

1 mistake (1 point) □

2 or more mistakes/untestable (2 points) □

Total Orientation Score: ____ /2

**Part 3: Assessment of Attention**

**3a)** Ask the patient **“Please tell me the months of the year backwards, starting at December.”** Allow one prompt **“What is the month before December?”**

Achieves 7 or more months correctly (0 points) □

Starts but scores <7 months/refuses to start (1 point) □

Untestable (cannot start because unwell, drowsy or inattentive) (2 points) □

Total Attention Score: ____ /2

**Part 4: Acute Change or Fluctuating Course**

**4a)** Is there evidence of significant change or fluctuation in attention, cognition or any mental function including hallucinations in the last 2 weeks, evident in the last 24 hours?

No (0 points) □

Yes (4 points) □

Total Acute Change or Fluctuating Course Score: ____ /4

**4AT Total Score: ___ /12**

**4AT Assessment Complete:** **Yes** □ **No** □

**ATTENTION: Days of the Week Backwards**

**Ask the patient “Can you tell me the days of the week backwards, starting with Sunday?**”

No mistakes (0 points) □

1 mistake (1 points) □

2+ mistakes, no response or UTA (2 points) □

**Record the order in which the participant responds:**

*Comments:* _______________________________________________________________________

_________________________________________________________________________________

**DOTWB Total Score: ___ /2**

**DOTWB Assessment Complete:** **Yes** □ **No** □

**PSYCHOTIC FEATURES: Hallucinations and Delusions**

Elicit any hallucinations and/or paranoid thoughts by asking: **“Have you been seeing or hearing anything unusual?”; “Are you concerned about anything going on here?”**

No hallucinations or delusions present (0 points) □

Patient appears suspicious or hints at experience of hallucinations/delusions i.e., mild suggestion of psychotic features (1 point) □

Detailed recollection of abnormal perception or thoughts i.e., strong indication that hallucinations and/or delusions are present (2 points) □

**Psychotic Features Total Score: ____ /2**

**Psychotic Features Assessment Complete:** **Yes** □ **No** □

**SPEECH: Production/Comprehension Assessment**

*Speech production*: Can the patient speak in meaningful sentences? **Yes** (0) □ **No** (1) □

*Verbal comprehension:* Can the patient follow a simple command? **Yes** (0) □ **No** (1) □

**“Can you hold up four fingers for me, like this?”** (Demonstrate once if needed).

**Communication Total Score: ___ /2**

**Communication Assessment Complete:** **Yes** □ **No** □

**DISTRESS Assessment (QDAT)**

Observe patient for signs of distress (frowning, looking anxious or fearful, restless) and ask them: **“How are you feeling? Is anything bothering you?”**

Patient appears settled, lying/sitting in bed comfortably, no signs of pain or distress (0 points) □

Patient verbalises mild distress and/or appears mildly concerned or worried (e.g., furrowed brow) (1 point) □

Patient verbalises moderate distress and/or displays physical signs of distress (e.g.

restlessness) (2 points) □

Patient verbalises severe distress and/or is visibly in a state of distress throughout assessment, requiring ongoing reassurance or possible intervention (3 points) □

**Observational Distress Total Score: ___ /3**

**Observational Distress Assessment Complete:** **Yes** □ **No** □

**END OF PATIENT INTERACTION**

**AROUSAL Assessment**

Does the patient open their eyes in response to voice? **Yes** (0) □ **No** (1) □

Does the patient keep their eyes open throughout the interaction? **Yes** (0) □ **No** (1) □

Patient maintains eye contact through interaction:

All the time (0 points) □

Sometimes (1 point) □

Not at all (2 points) □

**Arousal Total Score: ___ /4**

**Arousal Assessment Complete:** **Yes** □ **No** □

**Total Score**

**Index Assessment Total Score: ___ /25**

**Assessment Complete:** **Yes** □ **No** □

| **Researcher name** | **Researcher signature** |
| --- | --- |
| **Date** ____/____/____ | |

**Additional Comments**

________________________________________________________________________________________________________________________________________________________________________________________________________________________________________________________________________________________

**Consideration of Adverse Events**

Adverse events were considered by the researcher.

**Signature:** ______________________

Tick to indicate if any events occurred during the assessment.

| **AE** | **Yes** □ | **No** □ |
| --- | --- | --- |
| **SAE** | **Yes** □ | **No** □ |
| **ADE** | **Yes** □ | **No** □ |
| **SADE** | **Yes** □ | **No** □ |

**If applicable, please refer to the Adverse Event Log, located in the Investigator Site File Volume II Section 5.2, for additional information regarding this event.**

**Appendix 3**. DSM-5 structured reference standard flowchart for delirium.

**Appendix 4**. Supplementary Figure S1. 4AT scores across assessments for 18 participants who recovered from delirium (i.e. grouped as 'no delirium') by their last assessment.

Notes: Participants' delirium status according to the DSM reference standard assessment is indicate by red squares (delirium), green diamonds (partial delirium) and blue circles (no delirium). The dashed horizontal line indicates the 4AT cutoff score for delirium (4AT > 3). Only two participants were grouped as having no delirium at the second assessment, and one of them was delirious again at the third assessment. Interestingly, 6 participants were grouped as having 'partial (or resolving) delirium' at the second assessment, and these participants were delirium negative at subsequent assessment(s).

**Appendix 5**. Supplementary Table S1. Reference and index assessment scores for assessments 1, 2, 3 and 4.

| **Assessment** | **Assessment 1**  *N= 120 (100%)* | **Assessment 2**  *N= 120 (100%)* | **Assessment 3**  *N= 103 (85.8%)* | **Assessment 4**  *N= 69 (57.5%)* |
| --- | --- | --- | --- | --- |
| **Reference Standard** Assessment | | | | |
| Three-Item Recall (short,term) (/3) (median (IQR)) | 1 (0, 3) | 1 (0, 2.25) | 0 (0, 2) | 0 (0, 2) |
| Three-Item Recall (long,term) (/3) (median (IQR)) | 3 (2, 3) | 3 (1, 3) | 2 (1, 3) | 2 (1, 3) |
| Orientation (/3) (median (IQR)) | 3 (2, 3) | 2 (2, 3) | 2 (1, 3) | 2 (1, 3) |
| Counting backwards from 20 (/4) (median (IQR)) | 4 (0, 4) | 4 (0, 4) | 4 (0, 4) | 4 (0, 4) |
| Vigilance Test (CASABLANCA) (/10) (median (IQR)) | 2 (1, 4) | 2 (0, 4) | 1 (0, 4) | 1 (0, 4) |
| Short DelApp (/8) (median (IQR)) | 5 (2, 7) | 4 (2, 7) | 5 (2, 8) | 4 (3, 7) |
| RASS (-5 to +4) (median (IQR)) | 0 (-2, 0) | 0 (-1, 0) | 0 (-1, 0) | 0 (-1, 0) |
| OSLA (/15) (median (IQR)) | 4 (1, 6.25) | 3 (1, 6) | 2 (0, 5) | 2 (0, 6) |
| DRSR-98 total (/46) (mean (SD)) | 18.3 (5.38) | 15.9 (6.13) | 13.8 (7.02) | 14.2 (6.70) |
| **Index Assessment** | | | | |
| 4AT (/12) (mean (SD)) | 8.6 (2.70) | 7.7 (3.08) | 7.0 (3.67) | 6.6 (3.76) |
| Q-DAT (/3) (mean (SD)) | 1.2 (1.26) | 0.9 (0.89) | 0.8 (0.87) | 0.8 (0.90) |
| Days of the Week Backward (/2)  (median (IQR)) | 2 (1, 2) | 2 (0.75, 2) | 2 (0, 2) | 2 (0, 2) |
| Psychotic Features (/2) (median (IQR)) | 0 (0, 1) | 0 (0, 1) | 0 (0, 1) | 0 (0, 1) |
| Speech and Language (/2) (median (IQR)) | 1 (0, 1) | 0 (0, 1) | 0 (0, 1) | 0 (0, 1) |
| Arousal (/2) (median (IQR)) | 1 (0, 2) | 1 (0, 2) | 0 (0, 2) | 0 (0, 2) |

Notes: Three Item Recall (short-term and long-term) assessment are each scored 0-3, higher scores indicate more memory impairment. Orientation to time, place and location, score range 0-3, higher scores indicate worse orientation. Counting backwards from 20 is scored 0-4, higher scores indicate worse attention. Vigilance test (CASABLANCA) (score range 0-10, higher scores indicate worse performance). DelApp (short version) is scored 0-8, higher scores indicate better attention. RASS = Richmond Agitation-Sedation Scale, score range -5 (unarousable) to +4 (combative). OSLA = Observational Scale of Level of Arousal, score range 0-15, higher scores indicate more abnormal level of arousal, incorporating both reduced and increased arousal. DRS-R98 = Delirium Rating Scale-Revised 98, total score range 0-46, higher scores indicate increased likelihood and severity of delirium. A strict cut-off for the DRS-R98 was not used, but rather the sub items informed the DRS-R98 flowchart. 4AT is scored from 0-12 and scores ≥4 indicate delirium. Days of the Week Backward is scored 0-2, with higher scores indicating worse performance. Psychotic features (elicited via two interview questions) is scored between 0 (no hallucinations or delusions) and 2 (strong indication of hallucinations and/or delusions). Speech and language (speech production and comprehension) is scored 0-2, higher scores indicate more impaired speech and language. QDAT = Quick Distress Assessment Tool, score range 0-3, higher scores indicate more severe distress. Arousal assessment (eyes open to voice, maintaining eye opening and eye contact throughout interaction) is scored 0-2, higher scores indicate more arousal impairment.
